# Effect of diagnosis related groups implementation on the intensive care unit of a Swiss tertiary hospital: a cohort study

**DOI:** 10.1186/s12913-018-2869-4

**Published:** 2018-02-05

**Authors:** Lionel Chok, Esther B. Bachli, Peter Steiger, Dominique Bettex, Silvia R. Cottini, Emanuela Keller, Marco Maggiorini, Reto A. Schuepbach

**Affiliations:** 1 Institute of Intensive Care Medicine, University Hospital Zurich, University Zurich, Raemistrasse 100, CH-8091 Zurich, Switzerland; 2 Department of Internal Medicine, Hospital Uster, Brunnenstrasse 42, CH-8610 Uster, Zurich Switzerland

**Keywords:** Diagnosis related groups, DRG, ICU admissions, Epidemiology, Switzerland

## Abstract

**Background:**

In 2013 the Swiss Diagnosis Related Groups ((Swiss)-DRG) was implemented in Intensive Care Units (ICU). Its impact on hospitalizations has not yet been examined. We compared the number of ICU admissions, according to clinical severity and referring institution, and screened whether implementation of Swiss-DRG affected admission policy, ICU length-of-stay (ICU-LOS) or ICU mortality.

**Methods:**

Retrospective, single centre, cohort study conducted at the University Hospital Zurich, Switzerland between January 2009 and end of September 2013. Demographic and clinical data was retrieved from a quality assurance database.

**Results:**

Admissions (*n* = 17,231) before the introduction of Swiss-DRG were used to model expected admissions after DRG, and then compared to the observed admissions. Forecasting matched observations in patients with a high clinical severity admitted from internal units and external hospitals (admitted / predicted: 709 / 703, [95% Confidence Interval (CI), 658–748] and 302 / 332, [95% CI, 269–365] respectively). In patients with low severity of disease, in-house admissions became more frequent than expected and external admission were less frequent (admitted / predicted: 1972 / 1910, [95% CI, 1898–1940] and 436 / 518, [95% CI, 482–554] respectively). Various mechanisms related to Swiss-DRG may have led to these changes. DRG could not be linked to significant changes in regard to ICU-LOS and ICU mortality.

**Conclusions:**

DRG introduction had not affected ICU admissions policy, except for an increase of in-house patients with a low clinical severity of disease. DRG had neither affected ICU mortality nor ICU-LOS.

**Electronic supplementary material:**

The online version of this article (10.1186/s12913-018-2869-4) contains supplementary material, which is available to authorized users.

## Background

The Swiss health care system was rated second best in a recent overall ranking [1]. However, it is in seventh place as one of the most costly, consuming over 10.7% of the Swiss gross national product [2]. Since costs for hospitalized patients kept growing by 3% yearly since 1995 [2, 3], transparency of health care costs and activity, as well as improved efficiency, have become necessary. As a potential remedy, the diagnosis related group (DRG) system was adopted from Germany and implemented in acute care in 2012 and intensive care units on January 1st 2013 [4–7]. Swiss-DRG regulates reimbursement of health care providers. The DRG system assigns patients to an individual DRG (group) based on: the patient’s epidemiological information, diagnosis, clinical features and procedures [8]. Patients in an individual DRG (group) show a similar course of disease, comparable length of (hospital) stay (LOS) and comparable requirement for treatment. Thus, expenditures should also be comparable [9–11], allowing linkage of a specific DRG (group) to reimbursement. The United States of America first adopted this system in 1984 followed by most European and developed countries [10, 12–14].

Aiming at improving efficiency, Swiss-DRG defines an optimal LOS for a given clinical condition (DRG group). Prolonged LOS assumes that the caregiver was inefficient, and shortened LOS that services were incomplete. Thus, the DRG system dictates a reduced daily reimbursement rate for out of range LOS. To avoid this mechanism, caregivers might discharge or transfer economically unprofitable patients to ambulant care or services provided by unrelated hospitals, ultimately increasing resource consumption and readmission rates [10, 15, 16]. For example, in France such DRG related shifts between private and public institutions, and towards ambulatory services, have been well documented for obstetrical patients [17]. In the United Kingdom DRG resulted in the admission of patients in need of low complexity surgeries to private clinics, but admissions for more complex cases to public services [18]. In contrast, in Germany no such side effects have been linked to the DRG system [19], potentially due to specific design features.

Although causality has not yet been established, these examples illustrate the unintended phenomenon of selection of profitable cases, and changes in patient flow between acute care units observed after DRG implementation [10]. The absence of a risk adjustment in the Swiss-DRG system may encourage non-tertiary hospitals to engage in risk selection by admitting low, rather than high-risk, patients for elective interventions [20]. The Swiss DRG system compensates somewhat for certain therapeutic complexities such as prolonged ventilation or renal replacement therapy. However, coverage may be insufficient in high cost patients as well as in patients requiring ICU care, but not DRG reimbursed ventilation or renal replacement therapy. Our hospital’s authorities did not request any change in ICU admission policy after Swiss-DRG introduction at our institution. Nevertheless, unintended mechanisms, could have led to changes in number and type of admission. A shift of potentially unprofitable patients from the private to the public sector, or from secondary to tertiary hospitals, (as was reported for obstetrical patients [17]) could also affect ICU admissions, as these patients often require short post intervention ICU stay. Alternatively, financial pressure could reduce the availability of free ICU beds and redirect patients with a low burden of disease to the ward, and patients with minimal survival potential towards palliative care and away from the ICU [21].

To date, no data is available, to our knowledge, on how DRG affects ICU admission policies and ICU outcomes.

In order to analyse how the introduction of the Swiss-DRG system affects ICU admission policies, we retrospectively analysed the number of admissions, ICU-LOS, and mortality in ICUs of a tertiary Swiss University Hospital.

The primary aim was to assess the amount of ICU admissions of in and out patients during the pre-DRG period (2009 to 2012) and to compare admission policies before and after the implementation of Swiss-DRG on January 1st 2013. We further investigated whether the Swiss-DRG affected ICU-LOS and ICU mortality.

## Methods

### Study design

This retrospective, single-centre, observational trial complies with the current version of the Declaration of Helsinki and the national legal and regulatory requirements. It has been approved by the Canton Ethics Committee (Kantonale Ethikkommission Zurich, Switzerland, KEK-ZH-Nr. 2014–0452). This study was conducted at the University Hospital Zurich (UniversitätsSpital Zurich, Switzerland; USZ), a Swiss, tertiary care, referral, teaching hospital. This 860 bed hospital is Switzerland’s largest institution in terms of annual admissions and delivers health care services for over 1.4 million inhabitants in and around the Canton of Zurich [22, 23].

### Study population

All patients hospitalised in ICUs in the USZ between January 1st 2009 and December 31th 2013 were included. In addition to the implementation of Swiss-DRG on January 1st 2013, ICU admission policies were also affected by the inauguration of a large intermediate care unit (IMC) in October 2013. Therefore, all patients admitted to the ICU after the IMC was established were excluded from analysis (Additional file 1: Figure S1). Patients admitted during the period October to December of the years 2009–2012 were also excluded to avoid potential seasonal bias.

### Data collection

Demographic data on patients admitted to ICUs are routinely recorded for quality control and mandatory data reporting to federal institutions. Since January 1st 2009, the treating physicians and nurses have been trained to accurately record and enter patients’ characteristics such as vital parameters into an electronic system (KISIM™, Cistec®, Switzerland). The data entered is controlled by the attending physician followed by a final approval by the clinical manager. From this data, an anonymized subset of data was extracted containing demographics and clinical information. It contained (i) gender, (ii) age in years (no date of birth), (iii) ICU-LOS, (iv) clinical gravity at the time of admission defined by the simplified acute physiology score (SAPS II Score [24]), (v) eventual occurrence of death during ICU stay, (vi) origin of the patient (in-patient or external patient i.e. patients first treated by a hospital other than the USZ).

### Study objectives

All primary endpoints have been predetermined and submitted to the Canton Ethics Committee before data extraction and analysis. The primary objective was to evaluate whether the number of patients with severe disease admitted from external hospitals increased significantly after the introduction of the DRG system. To dichotomize ICU patients into very sick and less sick individuals we used the simplified acute physiology score (SAPS II [24]) at a cut off of 40 [25–29].

Secondary outcomes included the analysis of the severity-adjusted ICU-LOS and severity-adjusted ICU mortality (occurrence of death during ICU stay) before and after introduction of the Swiss-DRG. For this purpose, data was stratified by using the SAPS II admission score in order to divide the population into groups with similar characteristics.

### Data analysis and statistics

Datasets were analysed using the NCSS 2007 (NCSS Statistical Software, Kaysville, UT, USA), SPSS 22 (SPSS Inc., Chicago, IL, USA) and StataCorp 2017 (StataCorp, Stata Statistical Software: Release 15. College Station, TX: StataCorp LLC, USA) software packages. These were used together with Excel and Publisher (Microsoft Office 2010, Microsoft Corporation, Redmond, WA, USA) for data editing and presentation. There was no missing data. Patient characteristics were represented with descriptive statistics. The heterogeneity between the groups was assessed using Chi-square and Kruskal-Wallis tests.

In order to be able to examine whether implementation of the Swiss-DRG affected patient demographics, we compared ICU admissions after DRG implementation to admissions forecasted based on data recorded between 2009 and 2012. Forecasting of time series were used, based on an exponential smoothing model (NCSS software), which allowed for calculation of expected admissions with 95% Confidence Intervals (CI) [30, 31]. Exponential smoothing has been described as a reliable method for forecasting of health care time series [31]. The weighting parameter (α) of 0.3 resulted in the smallest mean of the squared errors and was therefore used. Using this model, we assumed that the evolution of medical sciences and the health care system was continuous throughout the whole study period and was not subject to major structural, financial, or political changes other than DRG implementation. Given the short time series available, we also used a Poisson regression model (SPSS Inc.) and a linear regression analysis based on forecasts with Newey-West standard errors (Lag 1) (StataCorp) to test the robustness of conclusions drawn by the exponential smoothing model. The Poisson regression model adjusts for the binomial variable DRG year/no-DRG year (logarithmic link function: Log (Y) = β0 + β1X1 + β_2_X_2_, Y being the response variable ‘count of admissions’, X_1_ and X_2_ the explanatory variables ‘year of admission’ and ‘DRG status’ respectively, β _0_ the intercept, β _1_ and β _2_ the regression coefficient of the first and second variable, respectively). As a third model to test for changes upon DRG implementation, we conducted sensitivity analyses using forecasts with Newey-West standard errors (Lag 1) using Statistical Software Stata 15.0 (StataCorp, College Station, TX: StataCorp LLC, USA). Previous studies assessing ICU patients described central values of dispersion of the SAPS II score (mean and/or median) being around 40 [25–29]. We thus defined patients having a SAPS II < 40 as cases with low clinical severity at ICU admission, and those with a SAPS II ≥ 40 as high severity cases.

In accordance to previous studies, we stratified patients by SAPS II (a score describing the severity of disease) to compare mortality between groups [26, 29, 32, 33].

Continuous secondary outcomes were analysed by using the least squares linear regression analysis if data was normally distributed. For non-normally distributed data, we used the nonparametric bivariate Spearman’s rank correlation. To test for trends by regression methods we entered SAPS strata into the model as a continuous variable. Results were considered significant at *P*-values of < 0.05 or if values were outside the 95% CI.

For comparisons of groups of continuous, non-normally distributed variables, we used the nonparametric Kruskal-Wallis and Man-Whitney tests and parametric one-way analysis of variance (ANOVA) and Dunnett t-tests. Binomial data (dichotomous outcomes, categorical variable) was analysed with the Chi-square test or Fisher’s exact test when cells had an expected frequency of > 5 and differences between the groups were weighted by the variance analysis.

## Results

### Characteristics of the population

Between January 1st 2009 and December 31, 2013, a total of 23′107 patients were admitted to the ICUs of our institution. From these, admissions occurring October to December were removed from analysis (*n* = 5876) in order to prevent bias caused by structural changes (inauguration of a 30 bed IMC in October 2013) and seasonal bias (Additional file 1: Figure S1). Consequently, 17,231 patients entered analysis (Table 1). Patients were stratified according to the year of admission, the type of admission (in-house versus external), and the clinical severity of disease (SAPS II).

**Table 1:** Baseline characteristics according to the year of admission

Year of admission	All	2009	2010	2011	2012	2013
Demographics						
Nbr. of Patients (% of total population)	17,231	3450 (20)	3498 (20.3)	3437 (19.9)	3427 (19.9)	3419 (19.8)
Age, median (IQR)	61 (47–72)	61 (46–71.25)	61 (47–71)	62 (48–72)	62 (49–73)	61 (48–71)
Male (%)	10,729 (62.3)	2128 (61.7)	2133 (61)	2158 (62.8)	2132 (62.2)	2178 (63.7)
In-house and external admission						
In-house admissions (%)	13,492 (78.3)	2761 (80)	2744 (78.4)	2668 (77.6)	2638 (77)	2681 (78.4)
External admissions (%)	3739 (21.7)	689 (20)	754 (21.6)	769 (22.4)	789 (23)	738 (21.6)
SAPS II Score < 40 and ≥40						
Patients with SAPS II < 40 (%)	13,058 (75.8)	2843 (82.4)	2651 (75.8)	2619 (76.2)	2537 (74)	2408 (70.4)
Patients with SAPS II ≥ 40 (%)	4173 (24.2)	607 (17.6)	847 (24.2)	818 (23.8)	890 (26)	1011 (29.6)
In-house and external admission according to SAPS II Score						
In-house patients with SAPS II < 40 (%)	10,590 (61.5)	2317 (67.2)	2180 (62.3)	2111 (61.4)	2010 (58.7)	1972 (57.7)
In-house patients with SAPS II ≥ 40 (%)	2902 (16.8)	444 (12.9)	564 (16.1)	557 (16.2)	628 (18.3)	709 (20.7)
External patients with SAPS II < 40 (%)	2468 (14.3)	526 (15.2)	471 (13.5)	508 (14.8)	527 (15.4)	436 (12.8)
External patients with SAPS II ≥ 40 (%)	1217 (7.4)	163 (4.7)	283 (8.1)	261 (7.6)	262 (7.6)	302 (8.8)

### Admissions stratified by origin and clinical gravity of disease

**Fig. 1:**
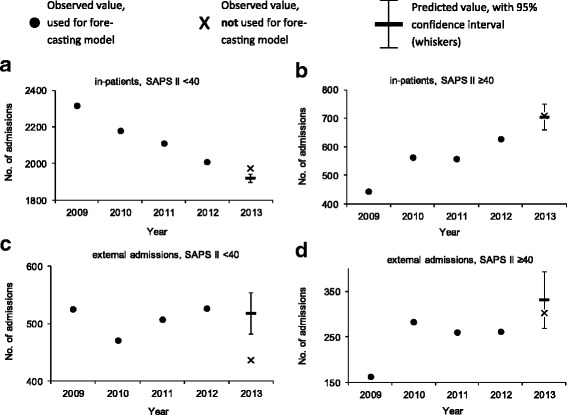
DRG affected admissions of patient with a low burden of disease. Patients were stratified by the year of admission, the origin of admission (in-house (**a**, **b**); from external hospitals (**c**, **d**)) and clinical severity at admission (SAPS II score < 40 (**a**, **c**); SAPS ≥40 (**b**, **d**)). Observed admissions in 2013 (x) are considered significantly affected by DRG if outside the 95% CI (whisker) of predicted admissions for 2013 (-) based admissions observed 2009 to 2012 (•). Forecasted and observed admissions were 1919 (95% CI: 1898–1940) and 1972 in (**a**), 703 (95% CI: 658–748) and 709 in (**b**), 518 (95% CI: 482–554) and 436 in (**c**) and 332 (95% CI: 269–395) and 302 respectively in (**d**)

In order to test whether Swiss-DRG affected admission policy to ICUs in our institution we used a forecasting model. Admissions observed between 2009 and 2012 were entered into an exponential smoothing model to forecast the number of patients expected for admission in 2013 (Fig. 1). Admissions observed in 2013 were considered to have been significantly affected by DRG if the number of observed admissions was outside the 95% CI of the admissions predicted by the forecasting model. We observed significantly more in-house admissions of patients with a low SAPS II score (SAPS II ≥ 40; observed count 1972, 95% CI of predicted count 1898–1940; Fig. 1a) than predicted, and fewer admissions than predicted of patients form external hospitals with a SAPS II < 40 (observed count 436, 95% CI of predicted count 482–554; Fig. 1c). An alternative forecast model based on regression analysis with Newey-West standard errors supported all conclusions obtained by the exponential smoothing model (Additional file 1: Figure S2). In line with the forecasting models, a Poisson regression model supported Swiss-DRG-influenced reduction of patients admitted with a SAPS II < 40 from external hospitals (Additional file 1: Table S1).

Notably, over the observation period the amount of very sick patients (SAPS II ≥ 40) generally increased, regardless of the admission type (internal or external) (Additional file 1: Table S2).

### Correlation between clinical gravity and ICU-LOS

**Fig. 2:**
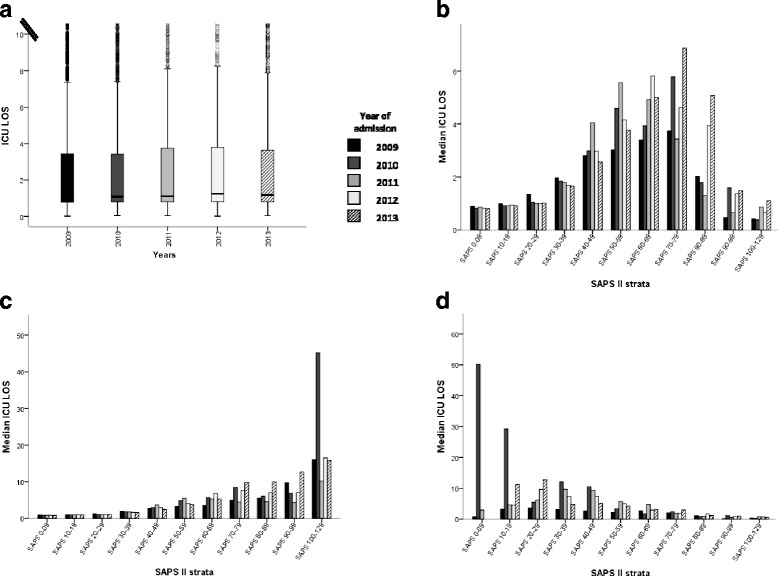
Relation between the clinical severity of disease at admission (SAPS II) and the ICU LOS. **a** Evolution of ICU LOS between 2009 and 2013. Comparison between the years (One-Way ANOVA) and to 2013 (Dunnett t-tests) n.s. **b** ICU LOS stratified by SAPS II and year of admission. ICU LOS in survivors (**c**) and no survivors (**d**) stratified by the year of admission and the clinical severity at admission (SAPS II Score)

Next, we addressed whether introduction of Swiss-DRG affected ICU-LOS. We found that ICU-LOS was not significantly different before and after DRG implementation (Fig. 2a). As we observed an increase in the number of very sick patients over time (Additional file 1: Table S2), we tested whether the severity of disease (SAPS II) affects ICU- LOS (Fig. 2b). We found median ICU-LOS to increase up to a SAPS II of 69, but to decrease with SAPS II higher than 70. As a result of this bell-shaped pattern, Spearman’s rank correlation coefficient (*r*_s_) only weakly correlated SAPS II and ICU-LOS (*r*_s_ = 0.37; Fig. 2b). This holds true for correlating SAPS and ICU-LOS for individual years, as well as for the whole study period (Additional file 1: Table S3). Using Fisher’s z transformation, we found the SAPS II correlated significantly stronger with the ICU-LOS in 2013 (*r*_s_ = 0.41) than in 2009 (*r*_s_ = 0.26), potentially indicating that disease severity gained influence on ICU-LOS over the years.

Median LOS was highest in patients with a SAPS II of around 70 and strata correlation revealed a positive SAPS II to LOS correlation up to a SAPS II of 69 (*r*_s_ = 0.39) but a negative correlation for sicker patients (*r*_s_ = − 0.30) (Table 2).

**Table 2:** The length of stay increased together with the clinical severity up to a SAPS II score of 69 and then decreases for even higher SPAS II scores

Year of admission	SAPS strata 1 to 7 (SAPS ≤69)		SAPS strata 8 to 11 (SAPS > 69)	
	Correlation coefficient (*r*_s_)	Independent correlation with 2013, *P* value ^1^	Correlation coefficient (*r*_s_)	Independent correlation with 2013, *P* value ^1^
All	0.39 **		−0.30 **	
2009	0.28 **	< 0.05 ^2^	−0.37 **	NS
2010	0.43 **	NS	−0.31 **	NS
2011	0.42 **	NS	−0.39 **	NS
2012	0.42 **	NS	−0.21 **	NS
2013	0.42 **		−0.30 **	

Patients were stratified by the year of admission and the clinical severity at admission (SAPS II score ≤ 69, SAPS II score > 69). The correlation of the SAPS II score and ICU LOS was considered significant if the *P* value was < 0.05 (*) using Spearman’s rank correlation. The correlation in 2013 was considered significantly affected by DRG if the independent correlation with the pre-DRG years was above 1.96 using Fisher’s z transformation

*NS* nonsignificant

**P value < 0.01

^1^The independent correlation with 2013 was calculated using Fisher’s z transformation. A *P* value < 0.05 meaning a significant difference between the correlations (the null hypothesis of equal correlation being rejected)

^2^The relationship between the SAPS and the LOS is significantly stronger in 2013 (*r* = 0.419) than in 2009 (*r* = 0.275)

We hypothesized that the bell-shaped relation between ICU-LOS and disease severity (SAPS II) could be explained by two factors driving short ICU-LOS: low burden of disease (low SAPS II) in ICU survivors, and high burden of diseases (high SAPS II) in ICU fatalities. To test for this, we stratified our study population into ICU survivors and ICU fatalities. In ICU survivors, the Spearman’s rank correlation positively correlated SAPS II and ICU-LOS (*r*_s_ = 0.40, *P* <  0.01) (Fig. 2c). This holds true for the whole study population as well as patients stratified by year, with the correlation being strongest in 2013 (Additional file 1: Table S4).

Inversely, in non-survivors the SAPS II and the ICU-LOS correlated negatively (*r*_s_ = − 0.40, *P* <  0.01) (Fig. 2d). This is again true for individual years as well as for the whole study population, with the correlation being strongest in 2013 (*r*_s_ = − 0.46) (Additional file 1: Table S4).

### ICU mortality before and after DRG introduction

**Fig. 3:**
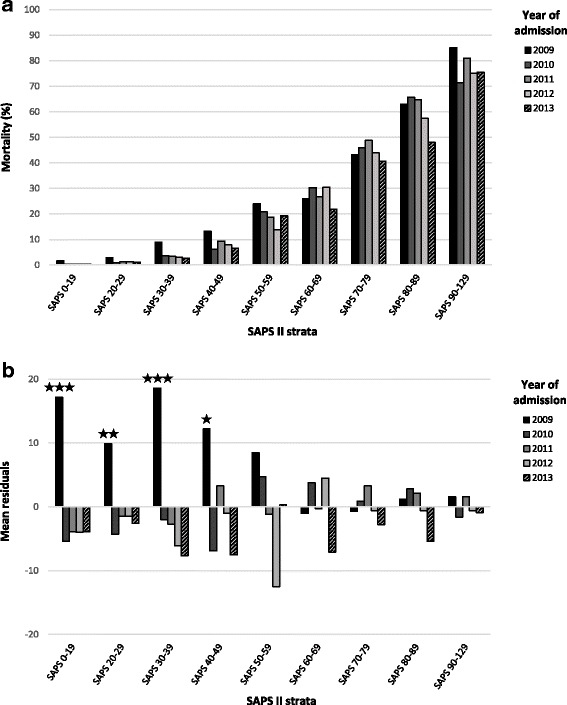
DRG did not increase the ICU mortality. **a** Intensive care unit mortality according to the year of admission and stratified by the clinical severity at admission (SAPS II score). **b** Mean residuals of Chi-square test assessing independency of ICU mortality and years 2009 to 2013, stratified by the clinical severity at admission (SAPS II score); asterisk refers to significant mortality difference with the other years. *P* < 0.5 (*), < 0.01 (**) < 0.001 (***)

Next, we addressed whether mortality changed over the years and especially in 2013, the year of Swiss-DRG introduction. Since the severity of disease increased over the study period (Additional file 1: Table S2) we not only stratified the study population by the year of admission, but also by severity of disease. In order to apply a Pearson Chi-square test to our data set, we needed to pool the few patients with low burden of disease (SAPS 0–19) as well as very sick patients (SAPS 90–129), while all others were grouped by increments of 10 SAPS II points (Fig. 3a).

Overall, mortality remained stable over the period analysed (Fig. 3b). However, there was a significant excess mortality in 2009 in patients with low burden of disease (SAPS II < 50). Although not significant, mortality was lowest in 5 of the strata in 2013, the year of Swiss-DRG introduction, not consistent with Swiss-DRG driving ICU mortality.

## Discussion

This study analysed the effect of the Swiss-DRG introduction on the number of admissions, the clinical gravity, the length-of-stay, and the mortality of patients admitted to a referral University hospital. We only detected minor effects of Swiss-DRG, especially on admission policies. For example, an increase of in-patients with a low clinical severity of disease, but fewer admissions of such patients from external hospitals (Fig. 1) were observed. The movement of patient populations are potentially linked, as bed availability in ICU is limited and the demand high. Importantly, other than a shift in admission policies, DRG did not affect ICU-LOS or mortality; in contrast, there was a trend towards reduced mortality after implementation of Swiss-DRG (Fig. 3).

Previous studies have linked DRG implementation to clinical outcomes such as LOS, mortality, quality of care, and patient satisfaction [10]. Whether our finding of reduced admissions of patients with low burden of disease from external hospitals (Fig. 1c) can be attributed to the Swiss-DRG system remains controversial. Shifts of low risk patients away from public referral centres have been reported [18]. However, the German DRG system which was used as a foundation for the Swiss-DRG, was reported to prevent such shifts [19].

The increase of in-house ICU admissions with low SAPS II score remains to be clarified (Fig. 1a). Firstly, risk selection by health care providers after moving to DRG-based reimbursement cannot fully be excluded. Of note, SAPS II score does not fully address all patients’ diagnoses, as relevant comorbidities such as diabetes mellitus or chronic obstructive pulmonary disease are not captured by this score. Further studies are needed to address whether the referral of patients with comorbidities (not captured by SAPS II) to tertiary public hospitals for procedures or diagnostics explains the increase of internal admissions with low SAPS II. Hence, one is tempted to speculate that under Swiss-DRG admission of high-risk, low profit cases after interventions or procedures increased in our tertiary hospital.

Hypothetically, due to the overtime increasing ICU bed constraints, the observed reduction of external referrals may also have led to the increase of in-house admissions by freeing beds for these patients.

Uncontrolled admissions, such as referrals from the emergency room or from non-ICUs units, however, are less susceptible to influence by a DRG-based remuneration. Indeed no decrease in in-house admissions was observed.

Over the 5-year study period, we identified a constant increase in admissions of patients with a high clinical severity (Fig. 1b, d) which was not further accentuated by Swiss-DRG implementation. This clearly shows that financial incentives independent of Swiss-DRG implementation optimized ICU admission policies. Increasingly non-ICU dependent patients were referred to cheaper institutions such as IMCs. Although we did not observe Swiss-DRG-related optimization, it may well occur with delay. Since the end of 2013, we have observed a massive decrease in ICU admissions of patients with a low burden of disease (not shown). We are not able to analyse whether this was a delayed effect of DRG. Since after October 2013 a large and continuous increase in IMC bed availability occurred in our institution, which could possibly explain our observations. Since specialized treatments are centralized in tertiary hospitals, changes in in-house or external admissions of patients with high severity of disease are unlikely to occur as a result of DRG implementation.

In Swiss acute care units LOS decreased continuously from 9.1 to 6.7 days during the previous decade, independent of DRG [2]. Thus optimisation already occurred long before Swiss-DRG was introduced. Whether DRG further reduces LOS and potentially ICU-LOS is controversial. A Swiss multicenter post-hoc study outside the ICU setting linked Swiss-DRG to shorter LOS [34], whereas two subsequent prospective analyses could not confirm optimisation [35, 36]. Similarly, in Germany, DRG had no detectable effect on LOS [19, 37]. We were also unable to directly link Swiss-DRG to ICU-LOS. Explanations included: (i) there is no such link, or (ii) the link has been obscured by many other influences and thus there is a lack of (statistical) power in our study. We found that variables affecting ICU-LOS included the burden of disease and mortality which, as reported, were interdependent [24]. We therefore dissected mortality and ICU-LOS and improved the correlation between SAPS II and LOS. Notably, we found this correlation to be negative in ICU non-survivors, likely because death reduces the risk for prolonged ICU-LOS. When the impact of DRG was tested again in this stratified set of data (Additional file 1: Table S4), SAPS II to ICU-LOS correlations were strongest in 2013. This is consistent with ICU-LOS which is driven by severity of disease and not logistic issues in ICU services and speculatively with more efficient end of life strategies in ICU non-survivors. In other words, there is some evidence, that Swiss-DRG enforces more rational, and faster, decisions as to whether to admit a patient to ICU and a patient’s need to stay in ICU.

Whereas improving efficiency is warranted, effectiveness (e.g. survival of the patient) is the ultimate goal of health services. We found mortality to decrease over the study period (Fig. 3) and Swiss-DRG to possibly coincide with an improved reduction of the ICU mortality. Our results confirm previous studies from USA, Australia and New Zealand, which overall described a significant fall in mortality over the past years [38, 39]. Similar to our conclusions, previous work from the US demonstrated no negative effects of DRG on mortality [40, 41] in general. Swiss data in patients with pneumonia [34] further confirms this conclusion. Whether indeed Swiss-DRG does not negatively affect ICU mortality remains to be clarified by additional research, since to our knowledge we are the first to provide such data.

Several limitations apply. First, the retrospective aspect of the analysis does not allow a full understanding of the mechanisms leading to the observed changes in clinical severity (SAPS II Score) and type (internal, external) of admission after DRG introduction. Although we assumed that no administrative and financial factors other than the DRG introduction have occurred, the number of beds available, the prevalence of epidemics, and administrative pressures are not addressed in this study. Secondly, our data might not be representative since the months October to December were excluded from the data analysed, due to structural changes in acute care provision in October 2013. Third, the data set without integration of clinical information (Diagnoses, comorbidities) excluded explorative analyses allowing for understanding in the shifts in admission policies. Last, the analysed period under DRG was short and effects might only become effective after prolonged time.

## Conclusion

To our knowledge, we present a first set of data on how Swiss-DRG influences our practices in Swiss ICUs. We believe that the relatively large data set and the design testing for an additional impact of Swiss-DRG, in an evolving system, are the primary strength of our analysis. In contrast, major drawbacks include the retrospective study design, exclusion of the months October to December, recruitment from only one single university hospital centre, and the short time series available due to structural changes at our institution.

Our data supports minor shifts of patient flow, which may well be of benefit for referral hospitals. Our data however support conclusions drawn in other countries where DRG has not negatively impacted quality in ICU services.

## Additional file

Additional file 1: Figure S1. Patient inclusion flow chart. **Figure S2.** Linear regression analysis based on forecasts with Newey-West standard errors (Lag 1). **Table S1.** DRG reduces the number of external admission in patients with low severity of disease. **Table S2.** The number of in-patients and external admissions with a high clinical severity increased significantly from 2009 to 2012, whereas admissions of in-patients with a low severity decreased. **Table S3.** Clinical severity of disease (SAPS II) at admission and LOS was weakly but positively corre-lated. **Table S4.** SAPS II and ICU LOS correlated positively in survivors, negatively in patients not surviving ICU. (PDF 1326 kb)

## Abbreviations

ANOVA: One-way analysis of variance
CI: Confidence Interval
DRG: Diagnosis related groups
ICU(s): Intensive care unit(s)
ICU-LOS: LOS: Length-of-ICU-stay
IMC: Intermediate care unit
LOS: Length-of-stay
*r*_s_: Spearman’s rank correlation coefficient
SAPS II: Simplified Acute Physiology Score
SwissDRG: Swiss Diagnosis related groups
USZ: University Hospital Zurich
