# Supplementary material for: Effect of diagnosis related groups implementation on the intensive care unit of a Swiss tertiary hospital: a cohort study
Source: BMC Health Serv Res. 2018 Feb 5;18:84. doi: 10.1186/s12913-018-2869-4 (PMC5800035; doi:10.1186/s12913-018-2869-4)
Supplement: Additional file 1: Figure S1. — Patient inclusion flow chart. Figure S2. Linear regression analysis based on forecasts with Newey-West standard errors (Lag 1). Table S1. DRG reduces the number of external admission in patients with low severity of disease. Table S2. The number of in-patients and external admissions with a high clinical severity increased significantly from 2009 to 2012, whereas admissions of in-patients with a low severity decreased. Table S3. Clinical severity of disease (SAPS II) at admission and LOS was weakly but positively corre-lated. Table S4. SAPS II and ICU LOS correlated positively in survivors, negatively in patients not surviving ICU. (PDF 1326 kb) [file 12913_2018_2869_MOESM1_ESM.pdf]

Supplements Fig 1. Patient inclusion flow chart

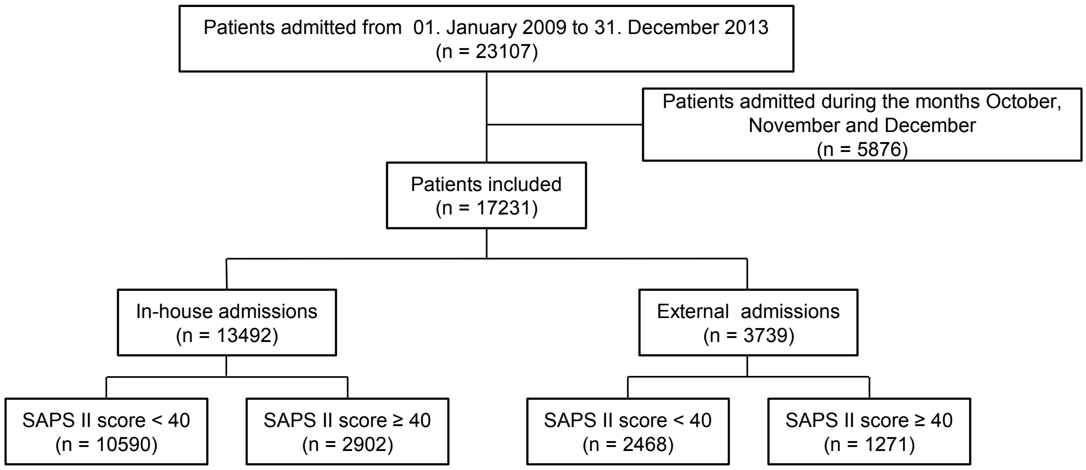

Supplements Fig 2. Linear regression analysis based on forecasts with Newey-West **standard errors (Lag 1).** Observed numbers of admissions and results of forecasts with 95% Confidence Intervals are shown.

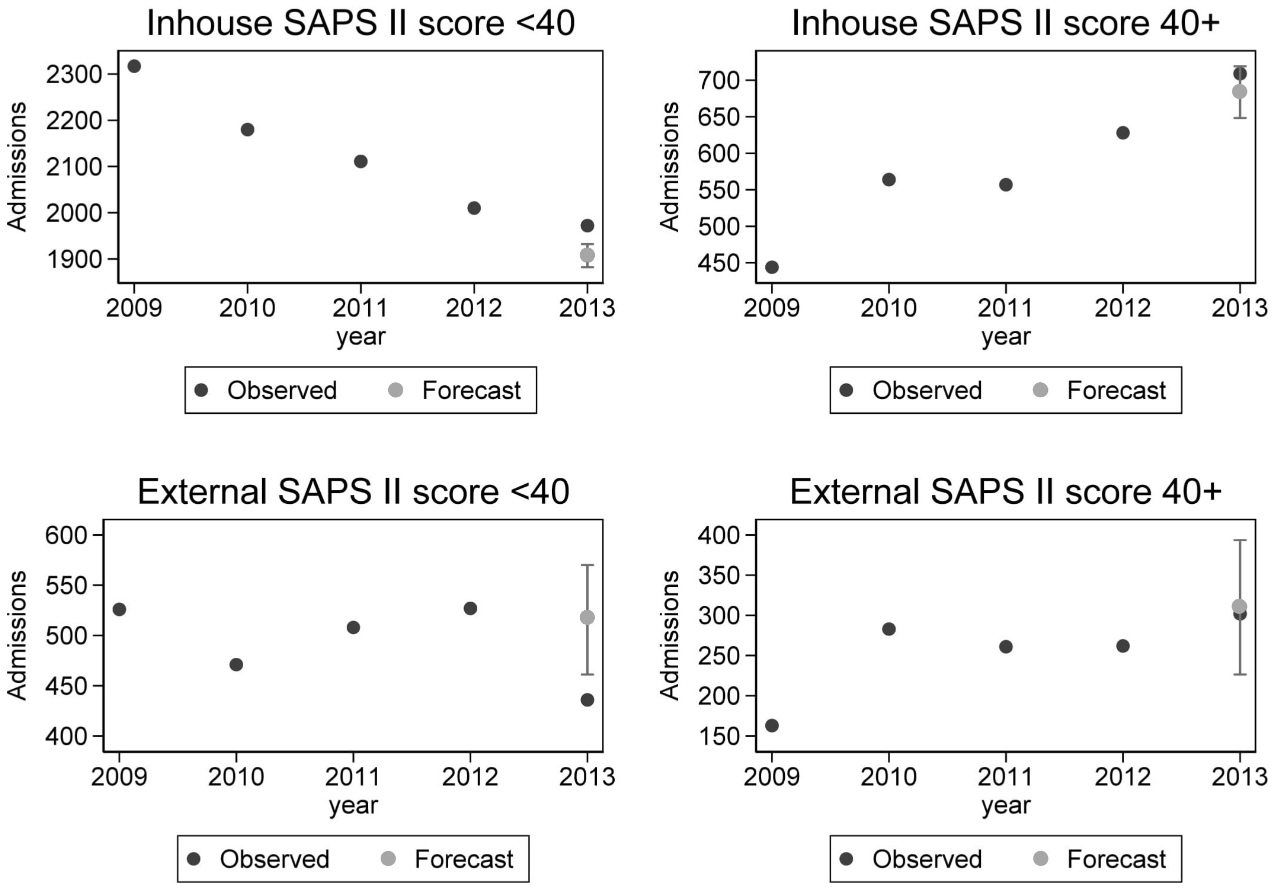

Supplements. Table 1. DRG reduces the number of external admission in patients with low severity of disease.

Patients were stratified by the origin of admission (in-patients (A,B) versus admission from external hospitals (C,D)) and clinical severity at admission (SAPS II score < 40 (A, C) and SAPS II score ≥ 40 (B, D)).

Observed admissions in 2013 are considered significantly affected by DRG (*) if the trend of admission in 2009 to 2013 (regression coefficient) is significantly other ($P < 0.05$) than the trend of admissions in 2009 to 2012[1].

**Type of admission**	**β_1, regression coefficient of variable 'DRG'** [3]	***95 % Wald Confidence Interval of the regression coefficient (lower limit / upper limit)***	***P value*** [2]
**A. In-house patients, SAPS II Score <40**	0.028	- 0.041 / 0.097	0.429
**B. In-house patients, SAPS II Score ≥40**	0.014	- 0.109 / 0.137	0.822
**C. External patients, SAPS II Score <40**	-0.173	- 0.314 / - 0.0308	**0.017**
**D. External patients, SAPS II Score ≥40**	-0.056	- 0.242 / 0.129	0.553

[1] See Supplements Table 2

[2] *P* value calculated using Wald Chi-square test

[3] Regression coefficient of the Poisson regression of the number of admissions against the explanatory variable DRG status (2009 to 2012 = no, 2013 = yes)

Supplements. Table 2. The number of in-patients and external admissions with a high clinical severity increased significantly from 2009 to 2012, whereas admissions of in-patients with a low severity decreased.

Poisson regression of the number of admissions against the explanatory variable 'year' (2009 - 2012) for the intern patients with a SAPS II Score <40 (A), intern patients with a SAPS II Score ≥40 (B), extern patients with a SAPS II Score <40 (C) and extern patients with a SAPS II Score ≥40 (D). Patients were stratified by the origin of admission (in-house (A,B) versus admission from external hospital (C,D)) and clinical severity at admission (SAPS II score < 40 (A, C) and SAPS II score ≥ 40 (B, D)).

**Groups of patients**	**β_1, regression coefficient of variable 'year'**	***95 % Wald Confidence Interval of the regression coefficient (lower limit / upper limit)***	***P value*** [1]
**A. Intern patients, SAPS II Score <40**	- 0.046	- 0.065 / -0.027	**< 0.001**
**B. Intern patients, SAPS II Score ≥40**	0.10	0.062 / 0.137	**< 0.001**
**C. Extern patients, SAPS II Score <40**	0.008	-0.03101 / 0.047	0.69
**D. Extern patients, SAPS II Score ≥40**	0.114	0.057 / 0.171	**< 0.001**

[1] *P* value calculated using Wald Chi-square test

Supplements. Table 3. Clinical severity of disease (SAPS II) at admission and LOS was weakly but positively correlated.

Patients were stratified by the year of admission. The correlation of the SAPS II score and the LOS was considered significant if $p < 0.05$ (*) using Spearman's rank correlation. To test whether strength of correlation was affected by DRG (year 2013) Fisher's z transformation was used.

	**SAPS II strata 1 to 11 (SAPS 0-129)**	
**Year of admission**	**Correlation coefficient (r_s)**	**Independent correlation with 2013** [1]
**2009 to 2013**	0.37 ***	
**2009**	0.26 ***	**< 0.05** [2]
**2010**	0.41 ***	NS
**2011**	0.38 ***	NS
**2012**	0.41 ***	NS
**2013**	0.41 ***	

NS = nonsignificant

*** *P* value < 0.001

[1] The independent correlation with 2013 was calculated using Fisher's z transformation. A *P* value < 0.05 meaning a significant difference between the correlations (the null hypothesis of equal correlation being rejected)

[2] The relationship between the SAPS and the LOS is significantly stronger in 2013 (r = 0.407) than in 2009 (r = 0.263)

Supplements. Table 4. SAPS II and ICU LOS correlated positively in survivors, negatively in patients not surviving ICU. Survivors and non-survivors were analyzed separately, then stratified by the year of admission. The correlation of the SAPS II score and the ICU LOS was considered significant if the *P* value was < 0.05 (*) using Spearman's rank correlation. The correlation in 2013 was considered significantly affected by DRG if the independent correlation with the pre-DRG years was above 1.96 or under –1.96 using Fisher's z transformation.

	**Survivors**		**Nonsurvivors**	
**Year**	**Correlation coefficient (r_s)**	**Independent correlation with 2013, *P* value** [1]	**Correlation coefficient (r_s)**	**Independent correlation with 2013, *P* value** [1]
**All**	0.40 ***		-0.40 ***	
**2009**	0.28 ***	**< 0.05** [2]	-0.23 **	**< 0.05** [3]
**2010**	0.44 ***	NS	-0.50 ***	NS
**2011**	0.42 ***	NS	-0.53 ***	NS
**2012**	0.44 ***	NS	-0.40 ***	NS
**2013**	0.44 ***		-0.46 ***	

NS = nonsignificant
** *P* value < 0.01
*** *P* value < 0.001
[1] The independent correlation with 2013 was calculated using Fisher's z transformation. A *P* value < 0.05 meaning a significant difference between the correlations (the null hypothesis of equal correlation being rejected)
[2] The relationship between the SAPS and the LOS is significantly stronger in 2013 (r = 0.439) than in 2009 (r = 0.284)
[3] The relationship between the SAPS and the LOS is significantly stronger in 2013 (r = -0.461) than in 2009 (r = -0.226)
